# High-throughput sequencing reveals genetic determinants associated with antibiotic resistance in *Campylobacter* spp. from farm-to-fork

**DOI:** 10.1371/journal.pone.0253797

**Published:** 2021-06-24

**Authors:** Shaimaa F. Mouftah, José F. Cobo-Díaz, Avelino Álvarez-Ordóñez, Menattallah Elserafy, Nehal A. Saif, Asmaa Sadat, Ayman El-Shibiny, Mohamed Elhadidy

**Affiliations:** 1 Biomedical Sciences Program, University of Science and Technology, Zewail City of Science and Technology, Giza, Egypt; 2 Department of Food Hygiene and Technology and Institute of Food Science and Technology, Universidad de León, León, Spain; 3 Center for Genomics, Helmy Institute for Medical Sciences, Zewail City of Science and Technology, Giza, Egypt; 4 Department of Bacteriology, Mycology and Immunology, Faculty of Veterinary Medicine, Mansoura University, Mansoura, Egypt; 5 Faculty of Environmental Agricultural Sciences, Arish University, Arish, Egypt; Zhejiang University, CHINA

## Abstract

*Campylobacter* species are one of the most common causative agents of gastroenteritis worldwide. Resistance against quinolone and macrolide antimicrobials, the most commonly used therapeutic options, poses a serious risk for campylobacteriosis treatment. Owing to whole genome sequencing advancements for rapid detection of antimicrobial resistance mechanisms, phenotypic and genotypic resistance trends along the “farm-to-fork” continuum can be determined. Here, we examined the resistance trends in 111 *Campylobacter* isolates (90 *C*. *jejuni* and 21 *C*. *coli*) recovered from clinical samples, commercial broiler carcasses and dairy products in Cairo, Egypt. Multidrug resistance (MDR) was observed in 10% of the isolates, mostly from *C*. *coli*. The prevalence of MDR was the highest in isolates collected from broiler carcasses (13.3%), followed by clinical isolates (10.5%), and finally isolates from dairy products (4%). The highest proportion of antimicrobial resistance in both species was against quinolones (ciprofloxacin and/or nalidixic acid) (68.4%), followed by tetracycline (51.3%), then erythromycin (12.6%) and aminoglycosides (streptomycin and/or gentamicin) (5.4%). Similar resistance rates were observed for quinolones, tetracycline, and erythromycin among isolates recovered from broiler carcasses and clinical samples highlighting the contribution of food of animal sources to human illness. Significant associations between phenotypic resistance and putative gene mutations was observed, with a high prevalence of the *gyrA* T86I substitution among quinolone resistant isolates, *tet(O)*, *tet(W)*, and *tet(32)* among tetracycline resistant isolates, and 23S rRNA A2075G and A2074T mutations among erythromycin resistant isolates. Emergence of resistance was attributed to the dissemination of resistance genes among various lineages, with the dominance of distinctive clones. For example, sub-lineages of CC828 in *C*. *coli* and CC21 in *C*. *jejuni* and the genetically related clonal complexes ‘CC206 and CC48’ and ‘CC464, CC353, CC354, CC574’, respectively, propagated across different niches sharing semi-homogenous resistance patterns.

## 1. Introduction

*Campylobacter* species (spp.), particularly *C*. *jejuni* and *C*. *coli*, are major foodborne pathogens and a leading cause of gastroenteritis worldwide [1]. *Campylobacter* is commonly prevalent in food producing animals, which act as a main reservoir for these pathogenic bacteria. Accordingly, humans become infected by the consumption of contaminated food of animal origin, especially undercooked poultry and unpasteurized milk and dairy products [2].

Campylobacteriosis is normally a self-limiting illness. Antimicrobial based therapy is only indicated for vulnerable patients with severe and invasive gastroenteritis. Macrolides and fluoroquinolones are the treatment of choice for the severe cases [3, 4]. Other antibiotics have been suggested as alternative therapeutic agents, such as tetracyclines, which are rarely used, particularly for children [5]. Moreover, meropenem, gentamicin, and telithromycin have revealed a strong *in vitro* activity against *Campylobacter* and might be considered as an alternative therapeutic option [3, 6]. Most of these antimicrobial agents have been used in veterinary practice as growth promoters and for the management of infections in food animals. Thus, the overuse and misuse of these antibiotics in clinical and veterinary settings have triggered the development and emergence of resistant *Campylobacter* isolates [7, 8]. Subsequently, with the widespread of resistance to numerous antibiotic classes, multidrug resistant (MDR) *Campylobacter* isolates are now being transferred from the food supply chain into the community [9, 10].

Several antimicrobial resistance (AMR) mechanisms have been well defined in *Campylobacter* spp., including target gene mutations, such as those in the *gyrA* and 23S rRNA genes, conferring resistance against fluoroquinolones and macrolides, respectively [5]. Likewise, mutations in *cmeABC*, the most common multidrug efflux pump in *Campylobacter*, play a vital role in promoting an increased resistance towards detergents and antimicrobial agents such as tetracycline, macrolides, and fluoroquinolones [11].

Previous studies on AMR mechanisms in *Campylobacter*, depended on low resolution techniques, have focused exclusively on the identification of only a handful of genes or point mutations [12, 13]. However, in recent years whole-genome sequencing (WGS) has improved the identification of various genetic determinants of phenotypic variation [14, 15]. WGS can reveal point mutations, mobile genetic elements and chromosomally encoded factors that play all together a vital role in resistance development, especially in MDR pathogens [16]. Moreover, WGS has enabled the upscaling of multi-locus sequence typing (MLST) schemes to core genome multi-locus sequence typing (cgMLST). The cgMLST is based on the analysis of a large number of genes shared by most members of a bacterial group, overcoming the low resolution of the conventional seven loci approach. Consequently, WGS can drive “One Health” surveillance through providing an unprecedented level of information that can be used to describe emerging trends of foodborne pathogens, linking animal and human health thus, consequently enhancing resistome surveillance [17].

In developing countries, including Egypt, campylobacteriosis cases are frequently reported [8, 18]. Yet, there is a paucity of data on the exact AMR patterns of *Campylobacter* along the food chain. Thus, it continues to be a challenge for food safety and public health in these countries to track the rapid emergence and geographic spread of AMR in *Campylobacter* [5, 10]. Accordingly, enhanced research efforts using WGS are needed in developing countries to identify factors affecting transmission and persistence of *Campylobacter* resistant clones in various environments and hosts. These efforts can consequently lead to the implementation of measures for containment of AMR.

The aim of this study is to investigate the mechanisms of resistance of 111 *Campylobacter* isolates, recovered in Cairo (Egypt) from cases of diarrheal disease in humans, commercial broiler carcasses, and dairy products. To achieve this aim, WGS was performed identifying genomic AMR determinants and genetic relationships between different lineages of *C*. *jejuni* and *C*. *coli*. Our findings contribute to the identification of transmission dynamics of antimicrobial resistance genes (ARGs) and their distribution by source.

## 2. Materials and methods

### 2.1. Bacterial isolates and culture conditions

A total of 111 *Campylobacter* isolates (90 *C*. *jejuni*, 21 *C*. *coli*) collected between 2017 and 2018 in Cairo, Egypt, from foods of animal origin (broiler carcasses; n = 30, milk and dairy products; n = 24) and fecal clinical samples (n = 57), were included in the study. The fecal strains had been isolated from patients suffering from diarrheal illness admitted to two different hospitals in Northern Giza, Egypt (S1 Table). All epidemiological information was available (e.g., date of sampling, hospital, age of the patient, clinical presentation). Patient names were anonymous.

Food strains had been obtained, following a stratified randomized sampling strategy, from different retail stores at the same location of the hospitals. Store names were de-identified, and the samples were re-numbered at the hospital. No permits were required for food samples collection as the food products were purchased from small-scale stores at the end of the production line. Each sample was packaged individually into a sterile container, marked, and transferred within 2–4 hours in an ice box for microbiological analysis.

Isolation of *Campylobacter* isolates from food matrices was performed according to the ISO 10272–1 (Selective Enrichment Method). Fecal clinical samples were inoculated on modified charcoal cefoperazone deoxycholate agar. Plates were incubated for 48 h at 42°C under anaerobic conditions using AnaeroGen^™^ 2.5L sachets (Oxoid, UK). Confirmation of *Campylobacter* at the genus level was performed using PCR identification of the *16S rRNA* gene, as described elsewhere [19].

### 2.2. Antimicrobial sensitivity testing

Antimicrobial susceptibility testing was performed using Sensititre^®^ broth microdilution plates (Thermo Fisher Scientific, USA) following the manufacturer’s instructions. Each plate contained a panel of serial dilutions of six antimicrobial agents belonging to four antibiotic classes. These included the quinolones ciprofloxacin (CIP) and nalidixic acid (NAL), the macrolide erythromycin (ERY), tetracycline (TET), and the aminoglycosides streptomycin (STR) and gentamicin (GEN). Plates were sealed and incubated at 37°C for 48 h in microaerophilic conditions. The minimum inhibitory concentration (MIC) values were defined as the lowest concentration that inhibited bacterial growth. An isolate was considered MDR when it demonstrated resistance to antibiotics from at least three different classes [20].

MICs interpretive criteria for resistance to CIP (R>0.5 mg/L), TET (R>2 mg/L), and ERY (R>4 mg/L for *C*. *jejuni* and >8 mg/L for *C*. *coli*) were set according to the European Committee of Antimicrobial Susceptibility Testing (EUCAST) (https://eucast.org/clinical_breakpoints/ last accessed: 01/2020). STR resistance (R>4 mg/L) was interpreted according to the EUCAST epidemiological cut-off values (ECOFFs). On the other hand, the National Antimicrobial Resistance Monitoring System for Enteric Bacteria (NARMS, 2015) breakpoints were used to define resistance criteria for NAL (R>16 mg/L) and GEN (R>2 mg/L). The *C*. *jejuni* strain ATCC 33560 was used as a quality control.

### 2.3. Whole genome sequencing

Genomic DNA was extracted from bacterial cultures using the QIAamp DNA Mini Kit (QIAGEN, UK), according to the manufacturer’s instructions. DNA was eluted in 100 μl of elution buffer and quantified using a Nanodrop spectrophotometer. Libraries were prepared using NexteraXT kits (v2) and a 2 × 300 base paired-end sequencing was performed on the Illumina’s MiSeq sequencing platform.

### 2.4. Data availability

Raw sequence reads are available on the NCBI`s sequence read archive (SRA) under the BioProject PRJNA576513 (https://www.ncbi.nlm.nih.gov/bioproject/PRJNA576513). For quality control of the Illumina reads, the QCtool pipeline [21] was used. The reads were assembled *de novo* using SPAdes (version 3.8.0) [22]. Assembled contigs were archived on the BIGSdb web-based platform [23], enabling species identification and MLST characterization, with assignment of the isolates to sequence types (STs) and clonal complexes (CCs). STs are assigned based on the alleles of seven defined genetic loci, which altogether define the allelic profile. The STs are then grouped into CCs that represent lineages possibly originating from a common ancestor given that they share at least five out of the seven alleles [24, 25].

### 2.5. Antimicrobial gene identification

In silico resistome identification using the genome sequences of all *Campylobacter* isolates was performed using CARD [26] and ResFinder [27] databases. Moreover, PointFinder was used to detect chromosomal single nucleotide polymorphisms (SNPs) linked to resistance phenotypes [28].

Additionally, raw reads were aligned against the 23S rRNA gene sequence of *Campylobacter jejuni* NCTC 11168, using Bowtie2 [29, 30], and the obtained 23S rRNA gene sequences were uploaded to the ResFinder webpage (https://cge.cbs.dtu.dk/services/ResFinder/) to search for chromosomal mutations associated with AMR. Moreover, bam files obtained from Bowtie2 alignments were manually inspected using the Geneious software (https://www.geneious.com/) to confirm the percentages of aligned reads that conveyed A2074T and A2075G mutations along the three copies of the 23S rRNA gene per each *Campylobacter* genome.

The *cmeR-cmeA* intervening region (*cme* RAIVS), and the *cmeR*, *rplD*, and *rplV* gene sequences were extracted from the contigs files by blastn search [31], with a 80% identity cut-off, using the genomes of *C*. *jejuni* NCTC 11168 and *C*. *coli* CFSAN054106 as references for the corresponding genes. The obtained sequences were inspected using MEGAX software (https://www.megasoftware.net/) to identify potential mutations associated with antimicrobial resistance (AMR).

### 2.6. Localization of the AMR genes

Plasmid sequences were identified using Plasflow [32]. Outputs from previous analyses with the CARD and ResFinder databases, where the contig location of each antimicrobial resistance gene (ARG) was determined, were compared with outputs from Plasflow to determine the location of ARGs within plasmids.

### 2.7. Phylogenetic tree construction

A cgMLST analysis was performed using the Genome Profiler (GeP) software [33]. The genomes of *C*. *jejuni* NCTC 11168 and *C*. *coli* CFSAN054106 were used as reference for the cgMLST scheme construction and cgMLST alleles sequence determination for each analyzed genome. Polymorphic genes detected by using the GeP software, listed on the *allele_calling*.*txt* file, were concatenated using this file and the.*fas* files on the scheme_* folder, both outputs from the GeP software, with an in-house ruby script (https://github.com/JoseCoboDiaz/concat_cgMLST_genes). As a result, a fasta file containing all core genome polymorphic genes concatenated in a single sequence per genome analyzed was obtained. The concatenated fasta file was then employed to construct a phylogenetic tree based on sequence differences, instead of allelic differences commonly used other approaches. This avoids the principal bias of considering the same distance or similarity value for alleles that can have different sequence distance. The concatenated gene-by-gene fasta files, one for the *C*. *jejuni* genomes and another for the *C*. *coli* genomes, were aligned on MAFFT version 7 web [34], using default parameters (i.e., *Auto* strategy, 200PAM / k = 2 scoring matrix, 1.53 of gap opening penalty and 0.0 offset value). A phylogenetic tree was calculated on MAFFT version 7 web [34] using the Neighbor-Joining method for conserved sites, the Jukes-Cantor substitution model and 1,000 bootstrap as re-sampling value. The generated newick files were downloaded, read by using the *ape* R-package and employed together with files containing the genomes characteristics and the analysis results to make the plots using the *ggtree* package on R (version 3.6.1).

### 2.8. Statistical analyses

Chi-square tests were employed for all statistical tests using R (version 3.6.1), with *p-*values lower than 0.05 considered as significant.

### 2.9. Ethical approval

The study represents a retrospective study that involves genome sequencing of a historical strains collection. No patient data collection was involved in this study.

## 3. Results

### 3.1. *C*. *coli* showed an enhanced *in vitro* MDR compared to *C*. *jejuni*

The *C*. *jejuni* (n = 90) and *C*. *coli* (n = 21) isolates were tested against six different antimicrobial agents (see S1 Table for AMR profiles). The highest proportion of phenotypic resistance was exhibited against nalidixic acid (NAL^R^); 62.2% (56/90) for *C*. *jejuni* and 90.4% (19/21) for *C*. *coli*, with MIC ranges of 32–64 mg/L for both species. That was followed by ciprofloxacin (CIP^R^); 60% (54/90) for *C*. *jejuni* with MIC ranges of 2–32 mg/L and 85.7% (18/21) for *C*. *coli* with MIC ranges of 2–16 mg/L. Resistance against tetracycline (TET^R^) was observed in 45.5% (41/90) of *C*. *jejuni* and 76% (16/21) of *C*. *coli*, with MIC ranges for both species of 4–64 mg/L. Moreover, the MICs of erythromycin (ERY), streptomycin (STR), and gentamicin (GEN) were generally higher among *C*. *coli* than among *C*. *jejuni* isolates. A total of 42.8% (9/21) of *C*. *coli* were ERY^R^ (MIC ranges of 16–128 mg/L) compared to 5.5% (5/90) of *C*. *jejuni* (MIC ranges of 8–32 mg/L). While only one *C*. *jejuni* isolate was STR^R^ (MIC of 8 mg/L), five *C*. *coli* isolates (23.8%; 5/21) were STR^R^ with a MIC of 8 mg/L. Finally, none of the *C*. *jejuni* isolates were GEN^R^, unlike five *C*. *coli* isolates (23.8%; 5/21) which showed resistance with MIC ranges of 4–8 mg/L (Table 1).

**Table 1:** Antimicrobial resistance patterns of *Campylobacter* isolates collected from foods of animal origin and clinical isolates.

		*Campylobacter jejuni* (n = 90)			*Campylobacter coli* (n = 21)		
	Antimicrobial agent	Clinical isolate (n = 42)	Broiler carcass (n = 26)	Dairy products (n = 22)	Clinical isolate (n = 15)	Broiler carcass (n = 4)	Dairy products (n = 2)
**MDR**	**CIP/NAL/TET/ERY**	**2/42 (4.7%)**	**-**	**-**	**3/15 (20%)**	**1/4 (25%)**	**-**
	**CIP/NAL/TET/STR**	**-**	**-**	**-**	**-**	**1/4 (25%)**	**-**
	**CIP/NAL/TET/ERY/GEN**	**-**	**-**	**-**	**-**	**-**	**½ (50%)**
	**CIP/NAL/TET/ERY/STR/GEN**	**-**	**-**	**-**	**1/15 (6.6%)**	**2/4 (50%)**	**-**
Non-MDR	CIP/NAL	9/42 (21.4%)	7/26 (27%)	4/22 (18%)	1/15 (6%)	-	½ (50%)
	CIP/NAL/TET	15/42 (35.7%)	10/26 (38.5%)	6/22 (27.2%)	5/15 (33.3%)	-	-
	CIP or NAL/TET	-	2/26 (7.6%)	1/22 (4.5%)	1/15 (6.6%)	-	-
	CIP/NAL/ERY	-	-	-	1/15 (6.6%)	-	-
	NAL/ ERY	1/42 (2.4%)	-	-	-	-	-
	TET/ ERY	1/42 (2.4%)	-	-	-	-	-
	CIP/NAL/STR/GEN	-	-	-	1/15 (6.6%)	-	-
	TET	2/42 (4.7%)	1/26 (3.8%)	1/22 (4.5%)	1/15 (6.6%)	-	-
	ERY	-	-	1/22 (4.5%)	-	-	-
	STR	-	-	1/22 (4.5%)	-	-	-
**Total number of non-multidrug resistant isolates**		28/42 (66.6%)	20/26, (77%)	14/22 (63.6%)	10/15 (66.6%)	-	½ (50%)
**Total number of multi-drug resistant isolates**		2/42 (4.7%)	-	-	4/15 (26.6%)	4/4, (100%)	½ (50%)
**Total number of sensitive isolates**		12/42 (28.5%)	6/26 (23%)	8/22 (36.3%)	1/15 (6.6%)	-	-

Overall, the most common AMR pattern (44%, 49/111) among both *Campylobacter* species was (CIP and/or NAL+TET)^R^. This AMR pattern was exhibited by 50% (15/30) of the isolates from broiler carcasses, 47.3% (27/57) of the isolates from clinical samples and 29% (7/24) of the isolates from dairy products. On the other hand, STR^R^ was more prevalent among isolates from broiler carcasses (10%; 3/30), followed by isolates from dairy products (4.2%; 1/24), and finally isolates of clinical origin (3.5%; 2/57). On the contrary, ERY^R^ was more prevalent among isolates of clinical origin (15.8%; 9/57), followed by isolates from broiler carcasses (10%; 3/30), and isolates from dairy products (8.3%; 2/24) (Table 1 and S1 Table).

A total of 10% of the tested isolates displayed MDR phenotypes, which were more frequent among *C*. *coli* (42.8%; 9/21) than *C*. *jejuni* (2.2%; 2/90) isolates (*p* <0.05). A total of 13.3% (4/30) of the MDR isolates had been isolated from broiler carcasses, while 10.5% (6/57) were of clinical origin and 4% (1/24) had been isolated from dairy products (Tables 1 and 2).

**Table 2:** Association of antimicrobial resistance phenotypes and the molecular mechanisms of antibiotic resistance among MDR *Campylobacter* isolates.

Sample ID	Species	Isolation Source	ST/CC	AMR Profile	Chromosomal mutation	AMR determinants*		
7729	*C*. *coli*	Broiler carcass	ST827/CC-828	CIP/NAL/TET/STR	gyrA	*-*	*-*	*bla*_*OXA-61*_^***C***^
7704	*C*. *coli*	Clinical sample	ST830/CC-828	CIP/NAL/TET/ERY	gyrA, 23S rRNA	*ant(6)-Ia-3* ^***C***^	*tet(O)*^***C***^ *tet(W/N/W)*^***C***^	*bla*_*OXA-61*_^***C***^
7693	*C*. *coli*	Broiler carcass	ST1055/CC-828	CIP/NAL/TET/ERY	gyrA, 23S rRNA	*-*	*tet(O)*^***P***^, *tet(W/N/W)*^***P***^	*bla*_*OXA-61*_^***C***^
7696	*C*. *coli*	Clinical sample	ST827/CC-828		gyrA, 23S rRNA		*tet(O)*^***C***^	*bla*_*OXA-61*_^***C***^
7711	*C*. *coli*	Clinical sample	ST7951/NA		gyrA, 23S rRNA	*lnuC* ^***C***^,*ant(6)-Ia-3* ^***C***^	*tet(O)*^***C***^	*bla*_*OXA-61*_^***C***^
7686	*C*. *jejuni*	Clinical sample	ST5221/NA		gyrA, 23S rRNA, rpsL	*-*	*tet(O)*^***C***^, *tet(O)*^***P***^,	*-*
							*tet(32)* ^***P***^	
7689	*C*. *jejuni*	Clinical sample	ST50/CC-21		gyrA	*-*	*tet(O)*^***P***^	*bla*_*OXA-61*_^***C***^
7646	*C*. *coil*	Dairy products	ST1059/CC-828	CIP/NAL/TET/ERY/GEN	gyrA, 23S rRNA	*-*	*tet(O)*^***C***^	*-*
7691	*C*. *coli*	Broiler carcass	ST1058/CC-828	CIP/NAL/TET/ERY/STR/GEN	gyrA, 23S rRNA	*-*	*tet(O)-tet(W/N/W)*^***P***^	*bla*_*OXA-61*_^***C***^
7692	*C*. *coli*	Broiler carcass	ST1191/CC-828		gyrA, 23S rRNA, rpsL	*-*	*tet(O)* ^***C***^	*bla*_*OXA-61*_^***C***^
7700	*C*. *coli*	Clinical sample	ST1016/CC-828	CIP/NAL/TET/ERY/STR/GEN	gyrA, 23S rRNA	*ant(6)-Ia-3* ^***C***^	*tet(O)*^***C***^, *tet(W/N/W)*^***C***^	*-*

* C: Chromosomally mediated, P: Plasmid mediated.

NA: Not Assigned.

### 3.2. Association of antimicrobial resistance patterns with clonal lineages in *C*. *jejuni* and *C*. *coli*

**Fig 1:**
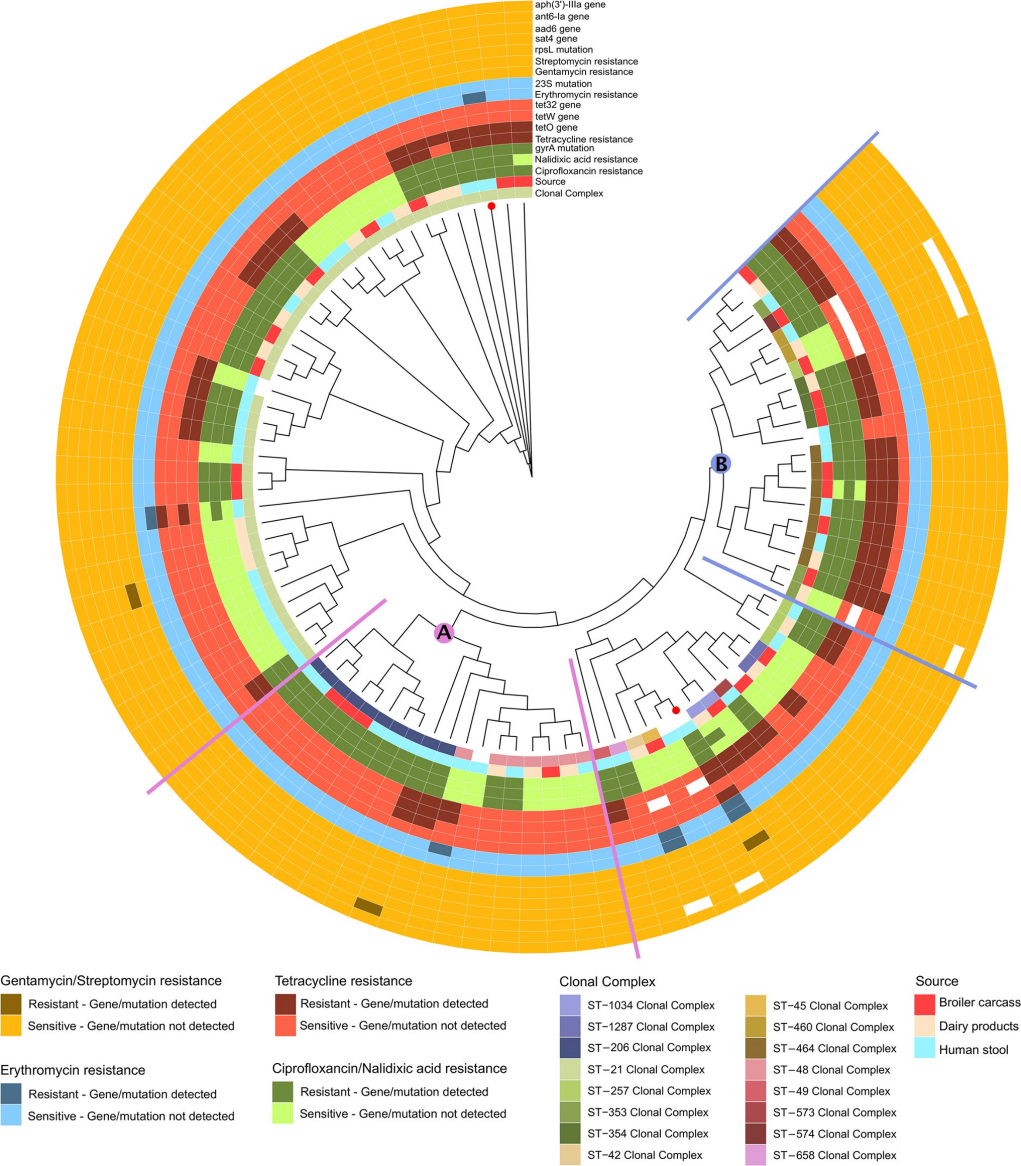
Phylogenetic tree for the 90 C. jejuni isolates using concatenated gene-by-gene alignments of core genes by Neighbor-Joining method, Jukes-Cantor substitution model and 1000 bootstrapping. A total of 320 core genes were obtained via cgMLST analysis. From inside-out, the first circle indicates the clonal complex according to MLST analysis. The second circle indicates the source of the isolate. The following circles indicate whether the isolates are resistant or sensible phenotypically to the antibiotics tested. MDR isolates are indicated by red dots. Isolates are grouped into clusters (A and B) based on nucleotide alignments. Mut.; mutation, res.; resistance.

The cgMLST analysis identified several genetic clusters mostly linked to CCs. The majority of the 90 *C*. *jejuni* isolates belonged to generalist CCs, among which CC21 (n = 36) was the most abundant, followed by genetically linked isolates belonging to CC206 (n = 10) and CC48 (n = 7) (Fig 1). Other specialist CCs that were genetically clustered were CC353 (n = 3), CC354 (n = 3), CC464 (n = 7), and CC574 (n = 1) (Fig 1). Moreover, isolates belonging to CC49, CC658, CC42, CC45, CC1034, and CC1287 showed partial genetic grouping (Fig 1).

The phenotypic AMR profiles observed were relatively homogeneous among genetically related lineages of *C*. *jejuni*, regardless of the isolation source. A total of 41% of the isolates exhibiting co-resistance against quinolones and tetracycline (CIP and/or NAL + TET)^R^ belonged to CC21 or the genetically linked (CC206 and CC48), while 38% were from the closely related lineages (CC464, CC353, CC354, CC574). On the other hand, the five ERY^R^ *C*. *jejuni* isolates were genetically distinct, belonging to CC21, CC48, CC42, and ST5221 with four of them being of clinical origin. Moreover, the only *C*. *jejuni* that was STR^R^ belonged to CC21 and was of clinical origin (Fig 1 and S1 Table).

**Fig 2:**
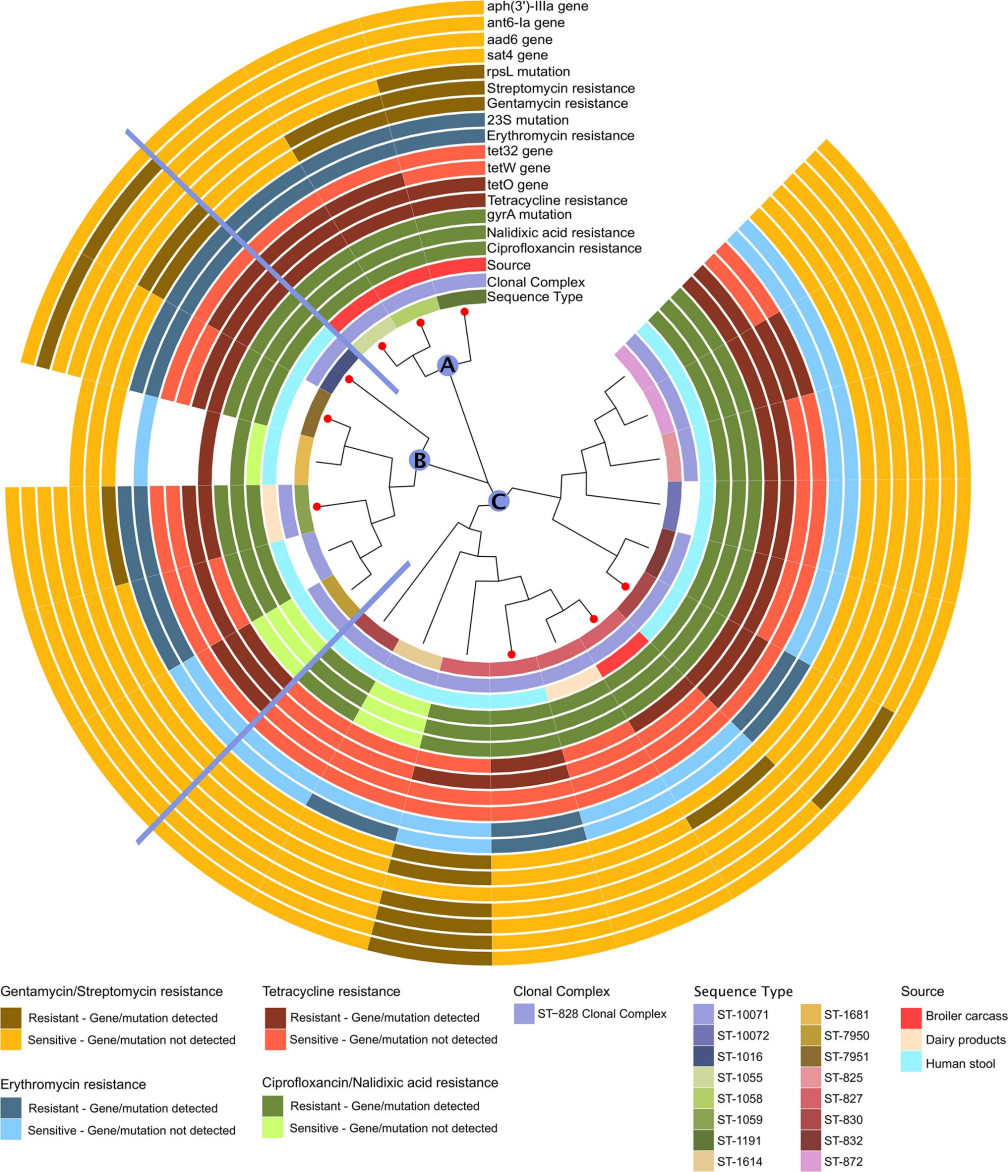
Phylogenetic tree for 21 *C*. *coli* isolates using concatenated gene-by-gene alignments of core genes by Neighbor-Joining method, Jukes-Cantor substitution model and 1000 bootstrapping. A total of 819 core genome genes were obtained via cgMLST analysis. From inside-out, the first circle indicates the clonal complex according to MLST analysis. The second circle indicates the source of the isolate. The following circles indicate whether the isolates are resistant or sensible phenotypically to the antibiotics tested MDR isolates are indicated by red dots. Isolates are grouped into clusters (A, B and C) based on nucleotide alignments.

Clonal divergence was less evident in *C*. *coli*, since 90.4% (19/21) of the isolates belonged to CC828 and two isolates were of an unassigned CC (ST-7951 and ST-1681) (S1 Table). Diversity within STs was analyzed using cgMLST, which revealed the clonal expansion of CC828 into three subclades: A, B, and C (Fig 2). Various AMR patterns were found in distinctive lineages within CC828, and, notably, they were partially similar to those showed by isolates from closely related STs. For instance, three isolates of subclade (A), all from broiler carcasses and belonging to CC828 but from different STs, were MDR. Two of these isolates exhibited resistance against all tested antibiotics, while the third was only sensitive to STR and GEN (Table 2). In subclade (B), 83% (5/6) of the isolates were of clinical origin and one isolate was from a dairy product, but all belonged to diverse STs. Three of these isolates were MDR exhibiting semi-homogenous resistance profiles (Table 2), while the other three exhibited different AMR profiles. Subclade (C) was the most diverse cluster within CC828 in terms of shared loci and STs. ST-827 seems to be the most successfully adapted ST. The later ST was the most abundant ST and was recovered from the three isolation sources, exhibiting semi-homogenous AMR patterns (Fig 2).

### 3.3. Unique AMR phenotype profiles among isolates of clinical origin

Two unique AMR profiles were exclusively displayed by *C*. *coli* isolates of clinical origin. The first profile (CIP+NAL+ERY)^R^, was exhibited by a ST10071-CC828 isolate (a newly assigned ST), while the second profile (CIP+NAL+STR+GEN)^R^ was displayed by a ST827-CC828 isolate (Table 1, Fig 2). Moreover, two unique resistance profiles were utterly found in *C*. *jejuni* isolates of clinical origin: (TET+ERY)^R^ exhibited by an isolate belonging to CC48, and (NAL-ERY)^R^ exhibited by an isolate belonging to CC21 (Table 1, Fig 1).

### 3.4. Prediction of putative resistance genetic determinants

The *bla*_*OXA-61*_ and *tet(O)* genes, associated with resistance to β-lactams and tetracyclines, respectively, were almost ubiquitous among both species. All OXA beta-lactamase genes were chromosomally encoded. A total of 78.8% (71/90) of the *C*. *jejuni* and 76.2% (16/21) of the *C*. *coli* genomes harbored *bla*_*OXA-61*_. On the other hand, four different OXA-variants (*bla*_*OXA-184*_, *bla*_*OXA-185*,_ *bla*_*OXA-465*_, *bla*_*OXA-448*_), were exclusively found in *C*. *jejuni* isolates. Moreover, all three components of a multidrug efflux tripartite system (*cmeA*, *cmeB*, and *cmeC*) were present in all isolates (Table 3, S2 Table).

**Table 3:** Total antimicrobial resistance rates with underlying molecular mechanism among tested *Campylobacter* isolates.

Antimicrobial (class)		Number of resistant isolates	Predicted AMR resistance and mechanism (acquired resistance or point mutation)	Number of positive for each genetic determinant
**Quinolones**	Ciprofloxacin	72	*gyrA* mutation	72
	Nalidixic acid	75	*gyrA* mutation	71
**Tetracyclines**	Tetracycline	57	*tet(O)*	55
			*tet(32)*	3
			*tet(W)*	16
**Macrolide**	Erythromycin	14	23S rRNA mutation	11
**Aminoglycoside**	Streptomycin	6	*sat-4*, *aad(6)*, *ant(6)Ia-3*, *aph(3’)IIIa*	1
			*rpsl* mutation	1
			*ant(6)Ia-3*	1
	Gentamicin	5	*sat-4*, *aad(6)*, *ant(6)Ia-3*, *aph(3’)IIIa*	1
			*ant(6)Ia-3*	1
			*rpsl* mutation	1

The *tet(O)* gene was significantly more abundant among *C*. *coli* (76%; 16/21) than in *C*. *jejuni* (47.7%; 43/90) (*p* <0.05). Whereas 87.5% (14/16) of the TET^R^ *C*. *coli* isolates harbored at least one of the known TET^R^ genes, two genetically distinct *C*. *coli* isolates lacked TET^R^ genes, but exhibited phenotypic TET^R^ with a MIC of 4 mg/L. On the contrary, other two genetically diverse *C*. *coli* genomes harbored TET^R^ genes, but did not show phenotypic TET^R^ (Fig 2). Furthermore, all *C*. *jejuni* isolates that were phenotypically TET^R^ carried at least one of the TET^R^ genes. However, two isolates which belonged to CC21 had plasmid mediated TET^R^ genes but didn’t show any phenotypic TET^R^ (Fig 1). The investigation of the localization of TET^R^ determinants in *C*. *jejuni* and *C*. *coli* revealed that *tet(O)* was either chromosomal- and/or plasmid-mediated. An apparent association of high TET MIC of 64 mg/L with the localization of the *tet(O)* gene on the chromosome was observed for both *C*. *jejuni* and *C*. *coli* (S1 and S2 Tables). In many isolates extra *tet(W/N/W)* and/or *tet* [32] genes were either co-localized on the chromosome or were plasmid-mediated (S2 Table).

With respect to ERY^R^, all nine ERY resistant *C*. *coli* isolates harbored the A2075G mutation in the 23S rRNA gene. However, one *C*. *coli* of clinical origin displayed the A2075G mutation in the 23S rRNA gene but didn’t show ERY^R^ phenotypically. Of the two ERY^R^ *C*. *jejuni* isolates, one isolate of clinical origin (ST5221) harbored the A2074T mutation in the 23S rRNA gene (MIC of 8 mg/L), while the other, from a dairy product (CC42), had the A2075G mutation but showed a higher MIC (32 mg/L). Conversely, three *C*. *jejuni* isolates of clinical origin (CC21, CC48 and MDR CC21) were ERY^R^ (MIC ranges of 8–32 mg/L) but did not harbor any known 23S rRNA gene mutations (Figs 1 and 2).

The 50S ribosomal subunit coding genes, which have been previously reported to be associated with phenotypic ERY^R^ in *Campylobacter*, were further investigated [35]. Specifically, the target sequences of the *rplD* gene in the amino acids spanning positions (63–74) of the L4 protein, or those of the *rplV* gene that code for the conserved ß-hairpin extension near the C-terminus of L22, were examined. Several amino acid substitutions and some insertions and deletions were identified in both ERY susceptible and resistant isolates, yet none was located in the target regions of the corresponding genes. Noteworthy, one L4 amino acid substitution (V184I) and one L22 amino acid substitution (A105M) were identified solely in ERY^R^ isolates (S2 Table).

The C257T transition in the quinolone resistance determining region (QRDR) of the *Campylobacter gyrA* gene, which results in a Thr86Ile substitution conferring resistance to quinolones, was identified in all except four isolates phenotypically exhibiting (CIP and/or NAL)^R^. These four isolates (*i*.*e*. three *C*. *jejuni*: one CC1033/dairy, one CC464/broiler carcass, and one CC21/clinical origin; one *C*. *coli* ST1681/clinical origin) showed phenotypic NAL^R^ with MIC of 32 mg/L (Figs 1 and 2).

The *CmeABC* efflux pump is transcriptionally repressed by *cmeR* by specifically binding to an inverted repeat (IR) region between *cmeR* and *cmeA*, termed as the *cme* RAIVS [*cmeR cmeA* intervening sequence] region (consensus sequence, TGTAATAAATATTACA (16-bp)). Mutations in the *cme* RAIVS site have been shown to impede the repression leading to an overexpression of *cmeABC* [36]. Further investigation of mutations revealed eight types of inverted repeat polymorphisms (IRPs) in the *cme* RAIVS site. The most frequent IRPs were found in *C*. *jejuni*, with 58.8% (53/90) of the *C*. *jejuni* isolates harboring Type 4 IRP and 6.6% (6/90) the Type 6 IRP. Unique *C*. *jejuni* isolates presented Type 1, Type 2, Type 3, and Type 5 IRPs while Type 7 and Type 8 were found only in *C*. *coli* (S2 Table). Compared with Type 0 representing the consensus sequence, isolates with Type 1 to Type 8 IRPs did not show any association with quinolone or ERY resistance (S2 Table, S1 and S2 Figs).

The association between phenotypic and genotypic aminoglycoside resistance (AG^R^) was only shown in two *C*. *coli* isolates. The isolates belonged to CC828, with one being a MDR isolate from broiler carcasses harboring a single base change in the *rpsL* locus (K43R) and the other being of clinical origin and harboring four AG^R^ determinants (i.e., *sat-4*, *aad(6)*, *ant(6)-Ia-3 and aph(3’)-IIIa*). Nevertheless, some inconsistencies were observed as four isolates (two *C*. *jejuni* and two *C*. *coli*) of clinical origin harbored *ant(6)-Ia-3* and a K43R mutation in the *rpsL* locus, but did not show phenotypic AG^R^. On the other hand, one *C*. *jejuni* and four *C*. *coli* isolates showed phenotypic AG^R^ despite not harboring any AG^R^ determinant (Figs 1 and 2, and Table 3).

Other ARGs were detected in low abundance. One *C*. *coli* isolate (CC828-clinical origin) harbored *cfr(C)*, which confers resistance to multiple antimicrobial classes including phenicols. Additionally, the *lnuC* gene, conferring resistance to lincosamides, was found in one MDR *C*. *coli* isolate (ST7951- clinical origin) (Table 2). Chloramphenicol and lincomycin were not included in our test panel, thus the correlation of resistance phenotypes and genotypes for these two genes could not be evaluated.

## 4. Discussion

Campylobacteriosis is the most commonly reported bacterial foodborne disease in both high- and low-income countries (1), thus escalation in AMR in *Campylobacter* is a major threat to human and animal health [37]. This is particularly critical in developing countries where antibiotic stewardship is challenging and regulations to govern the use of antibiotics in both clinical and veterinary settings are minimal [8, 18]. Accordingly, advanced research efforts are needed in developing countries to understand the factors affecting transmission and persistence of resistant *Campylobacter* isolates in various reservoirs and hosts.

Our data evidences a high prevalence of quinolones and tetracycline resistance rates among *Campylobacter* isolates. This observation, in addition to data recently published from Iran, another developing country [38], suggests that quinolones cannot be considered as an accepted empirical therapy for bacterial foodborne diarrhea, including that caused by *Campylobacter*. Moreover, our data reflects the inappropriate use of tetracyclines in veterinary settings, either as a growth promoter or for the treatment of domestic farm animals, which has decreased its clinical usefulness [39]. Equally worrying was the fact that 12.6% of the tested isolates were erythromycin resistant, since ERY is considered as the drug of choice for the treatment of clinical campylobacteriosis [40]. An apparent trend (~10%) towards MDR phenotypes in different niches, with the overall MDR rate in *C*. *coli* being higher than in *C*. *jejuni*, was evident, which agrees with previous observations [10, 41]. Altogether, this will render therapy more precarious and occasionally ineffective as by time all available drugs might not work as expected, especially in developing countries.

cgMLST revealed the existence of distinctive high-risk lineages that are commonly found in multiple niches disseminating AMR across the population. Among *C*. *jejuni*, CC21, together with the genetically linked CC206 and CC48, contribute with the majority of isolates exhibiting co-resistance against (CIP and/or NAL + TET), followed by the closely related lineages CC464, CC353, CC354, and CC574. Moreover, the majority of ERY^R^ *C*. *jejuni* were either CC21 or CC48, while STR^R^ *C*. *jejuni* belonged to CC21. Interestingly, unique AMR trends were observed exclusively among some *C*. *jejuni* isolates of clinical origin belonging to CC21 and CC48. Accordingly, this supports the potential of these lineages to persist, favoring zoonotic transmission and dissemination of resistance among different niches on a local level, thus frequently causing human disease as previously reported [9, 42]. Compared to *C*. *jejuni*, a greater proportion of *C*. *coli* isolates were (CIP and/or NAL + TET) and/or ERY resistant owing to the clonal expansion of CC828 across several niches. This observation agrees with findings from previous studies revealing the high frequency of specific mutations, and interspecies recombination, among strains belonging to *C*. *coli* CC828 [43].

Moreover, AMR prevalence among the isolates from broiler carcasses was often similar to that observed in isolates recovered from clinical cases, supporting the evidence that resistance patterns in foods of animal source may predict and contribute to AMR patterns in humans [7]. Indeed, consumption of undercooked poultry meat and unpasteurized dairy products is considered as a risk factor for domestically acquired resistant infections.

The main genetic determinant for quinolone resistance was the C257T transition in the QRDR of the *gyrA* gene, which is consistent with previous reports [44]. However, four isolates displayed phenotypic NAL^R^, but no mutations in *gyrA* gene were observed. This contradicts previous reports indicating that development of resistance in the absence of mutations in *gyrA* is rare [45]. Mutations in some putative drug efflux pumps that have been detected in *Campylobacter* might be responsible for this phenomenon, although their roles in quinolone resistance are to be determined yet [46]. On the other hand, the A2075G substitution in the 23S rRNA gene was the most prevalent genetic mechanism among ERY resistant isolates, corroborating a previous report that suggested a predominant role of this mutation in erythromycin resistance [47]. Our data is however in disagreement with the fact that this kind of mutation confers a high-level ERY^R^ (MIC>128 mg/L), as the MIC of ERY in resistant isolates ranged from 16 to 128 mg/L [48]. Among the two ERY resistant *C*. *jejuni* isolates, one harbored the A2074T substitution (MIC 8 mg/L), supporting previous studies which associated this mutation with resistance at low MICs among *Campylobacter* [5].

Remarkably, in the current study several substitutions were solely identified in ERY^R^ isolates. For example, “V184I” in L4 was identified in one of the tested *C*. *coli* isolates and “A105M” in L22 in one of the tested *C*. *jejuni* isolates, with MICs of 64 and 8 mg/L, respectively as previously reported [12]. Since those ERY^R^ isolates already harbored 23S rRNA gene mutations, these substitutions might have a little direct role in ERY^R^ in *Campylobacter* and cannot be unequivocally identified as an ERY^R^ mechanism. Further experimentation is needed for further elucidation in this regard.

Noteworthy, on a previous study using a stepwise selection process, mutations in the L4 and L22 genes were shown to occur earlier, while mutations in the 23S rRNA gene arose later. Thus, selection of mutations in the 23S rRNA gene seems to require prolonged times under sustained exposure to macrolides [49]. The abundance of alterations and mutations in the L4 and L22 genes among our tested isolates is an indicator of inappropriate use of macrolides in Egypt, which might lead to an escalation in the rate of erythromycin resistance in the upcoming years. This ultimately can lead to symptomatic relapse and treatment failure. In this regard, it is worth highlighting that resistance against erythromycin was more abundant among isolates of clinical origin.

The *bla*_*OXA-61*_ gene has been reported to confer resistance against beta-lactams in *Campylobacter* isolates [50]. Most of the tested isolates harbored the *bla*_*OXA-61*_ gene, which agrees with the findings of previous studies [10, 50, 51]. Nevertheless, *Campylobacter* is intrinsically resistant to beta-lactams. Consequently, they are not recommended for treating campylobacteriosis.

*Campylobacter* tends to acquire foreign AMR genes from other bacteria through horizontal gene transfer [10]. This was also evidenced in the current study, as *tet(O)*, *tet(W)* and *tet* [32] genes were dispersed along various lineages with the conservation of some TET^R^ genes on plasmids. This facilitates their spread into multiple genetic backgrounds, not only within the same species but also across different species and in multiple niches. Surprisingly, *C*. *coli* might have a yet unknown mechanism of tetracycline resistance, as two MDR isolates were tetracycline resistant despite not having any known tetracycline resistance determinant. This phenomenon could be however attributed to mutations in the *cmeABC* efflux pump, as previously reported [11].

The *lnuC* and *crfc(C)* genes were exclusively found among *C*. *coli* isolates from clinical origin, in agreement with the results of some studies reporting these two uncommon ARGs in *C*. *coli* isolates [10, 41]. The *crf(C)* is a novel MDR gene that confers resistance to five diverse antimicrobial classes in *Campylobacter* including phenicols, which are more often used in veterinary medicine rather than for treating clinical *Campylobacteriosis* in humans [52]. This can be imposing a selective pressure and leading to the emergence of new resistant isolates harboring this AMR determinant.

To conclude, this study reveals the potential of WGS for the in silico prediction of novel AMR mechanisms in *Campylobacter*. Moreover, by using cgMLST analysis, the current study has established a readily maintained local scheme for surveillance of *Campylobacter* isolates, which can be shared with other international and national laboratories using online databases. Thus, providing a more advanced microbial risk analysis. Additionally, cgMLST analysis revealed distinctive genotypes which are propagating between multiple niches in Egypt, denoting poor sanitary practices. The endurance of preexisting resistant *Campylobacter* clones at farm level and their eventual dissemination along the food chain can be the cause of human infections by MDR *Campylobacter*. Therefore, this study reflects the importance of implementing WGS to set prevention strategies and strict antibiotic stewardship in the Egyptian market, to combat the increasing threat of AMR. Our future studies will implement probabilistic source attribution from WGS data to facilitate source tracking of different MDR pathogens from farm-to-fork.

## Supporting information

S1 Fig Alignment of intergenic region of *Campylobacter jejuni* NCTC 11168 reference strain with five ERY^R^ *C*. *jejuni* isolates. (PPTX)

S2 Fig Alignment of intergenic region of *Campylobacter coli* CNM20070465 reference strain with nine ERY^R^ *C*. *coli* isolates. (PPTX)

S1 Table Antimicrobial susceptibility testing of 111 *Campylobacter* isolates against six different antimicrobial agents. (XLSX)

S2 Table Molecular characterization of antimicrobial resistance mechanism in 111 *Campylobacter* isolates. (XLSX)

S3 Table Comparison of frequency of Sequence Types (STs) in this study and those found in genomes from NCBI GenBank database. (XLSX)
