# Supplementary material for: High-throughput sequencing reveals genetic determinants associated with antibiotic resistance in Campylobacter spp. from farm-to-fork
Source: PLoS One. 2021 Jun 24;16(6):e0253797. doi: 10.1371/journal.pone.0253797 (PMC8224912; doi:10.1371/journal.pone.0253797)
Supplement: S1 Fig — (PPTX) [file pone.0253797.s001.pptx]

## Slide 1
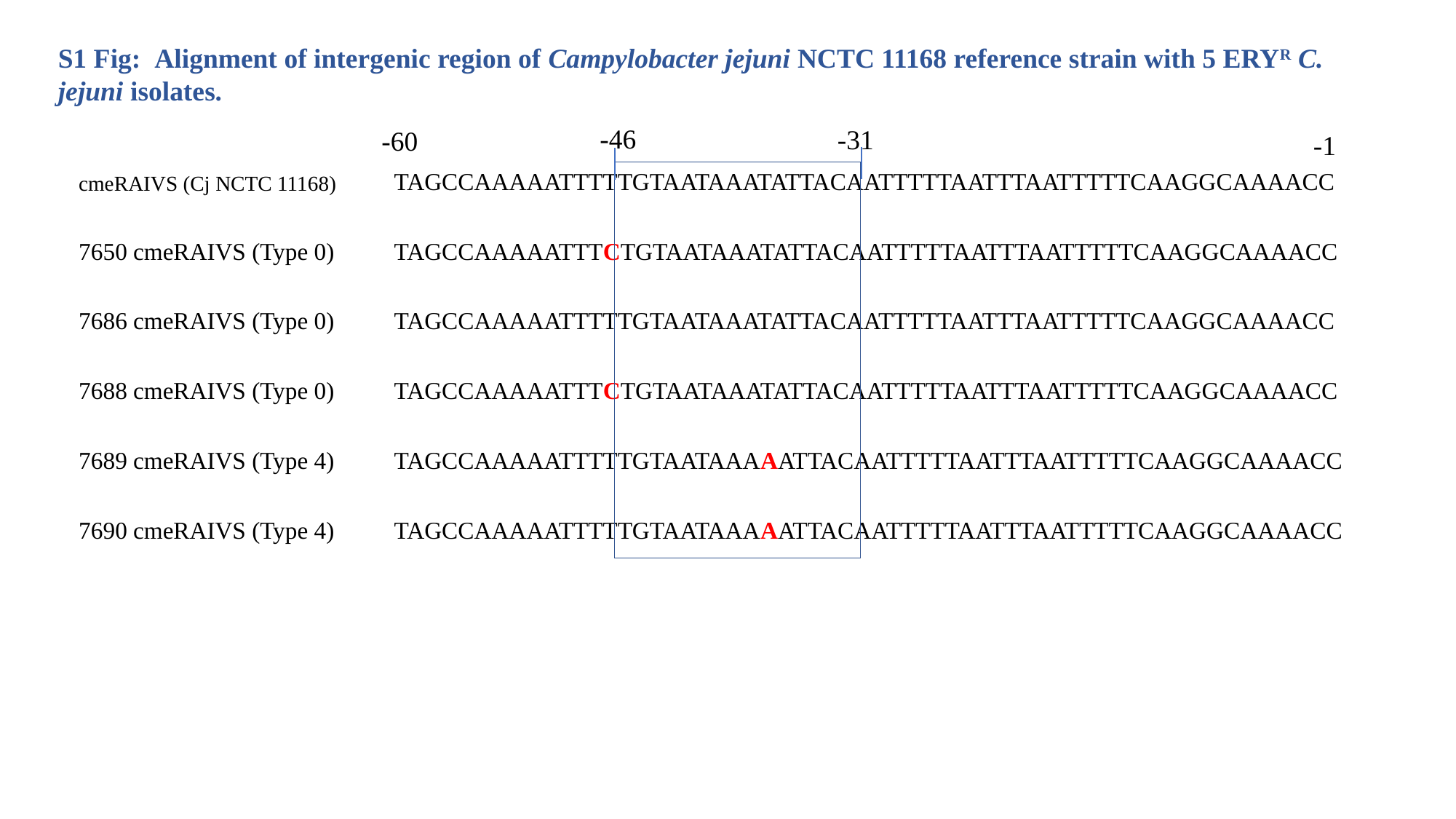

S1 Fig:  Alignment of intergenic region of Campylobacter jejuni NCTC 11168 reference strain with 5 ERYR C. jejuni isolates.
-46
-31
-60
-1
| cmeRAIVS (Cj NCTC 11168) | TAGCCAAAAATTTTTGTAATAAATATTACAATTTTTAATTTAATTTTTCAAGGCAAAACC |
| --- | --- |
| 7650 cmeRAIVS (Type 0) | TAGCCAAAAATTTCTGTAATAAATATTACAATTTTTAATTTAATTTTTCAAGGCAAAACC |
| 7686 cmeRAIVS (Type 0) | TAGCCAAAAATTTTTGTAATAAATATTACAATTTTTAATTTAATTTTTCAAGGCAAAACC |
| 7688 cmeRAIVS (Type 0) | TAGCCAAAAATTTCTGTAATAAATATTACAATTTTTAATTTAATTTTTCAAGGCAAAACC |
| 7689 cmeRAIVS (Type 4) | TAGCCAAAAATTTTTGTAATAAAAATTACAATTTTTAATTTAATTTTTCAAGGCAAAACC |
| 7690 cmeRAIVS (Type 4) | TAGCCAAAAATTTTTGTAATAAAAATTACAATTTTTAATTTAATTTTTCAAGGCAAAACC |
