# Supplementary material for: High-throughput sequencing reveals genetic determinants associated with antibiotic resistance in Campylobacter spp. from farm-to-fork
Source: PLoS One. 2021 Jun 24;16(6):e0253797. doi: 10.1371/journal.pone.0253797 (PMC8224912; doi:10.1371/journal.pone.0253797)
Supplement: S2 Fig — (PPTX) [file pone.0253797.s002.pptx]

## Slide 1
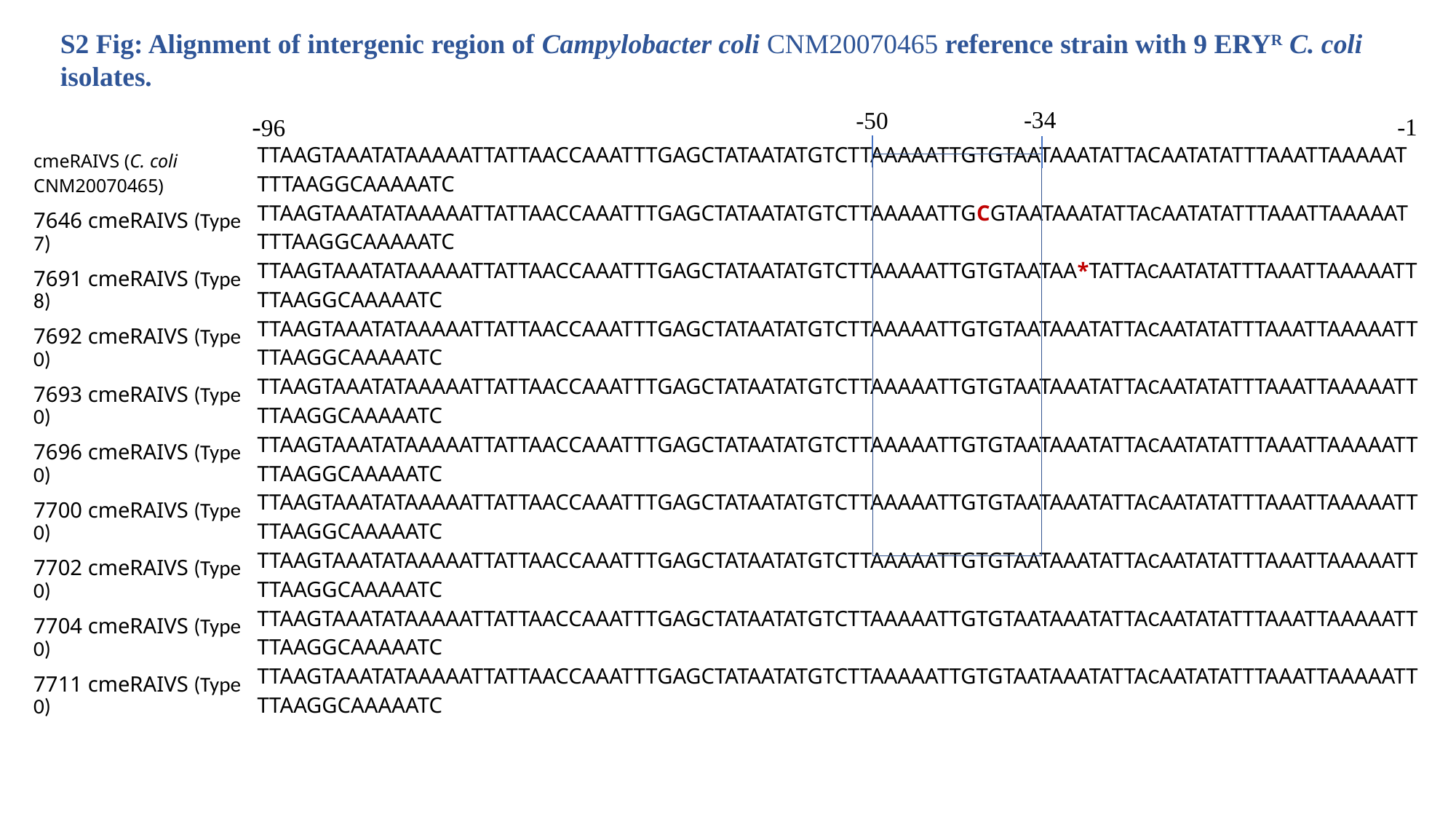

S2 Fig: Alignment of intergenic region of Campylobacter coli CNM20070465 reference strain with 9 ERYR C. coli isolates.
-34
-50
-96
-1
| cmeRAIVS (C. coli CNM20070465) | TTAAGTAAATATAAAAATTATTAACCAAATTTGAGCTATAATATGTCTTAAAAATTGTGTAATAAATATTACAATATATTTAAATTAAAAATTTTAAGGCAAAAATC |
| --- | --- |
| 7646 cmeRAIVS (Type 7) | TTAAGTAAATATAAAAATTATTAACCAAATTTGAGCTATAATATGTCTTAAAAATTGCGTAATAAATATTACAATATATTTAAATTAAAAATTTTAAGGCAAAAATC |
| 7691 cmeRAIVS (Type 8) | TTAAGTAAATATAAAAATTATTAACCAAATTTGAGCTATAATATGTCTTAAAAATTGTGTAATAA\*TATTACAATATATTTAAATTAAAAATTTTAAGGCAAAAATC |
| 7692 cmeRAIVS (Type 0) | TTAAGTAAATATAAAAATTATTAACCAAATTTGAGCTATAATATGTCTTAAAAATTGTGTAATAAATATTACAATATATTTAAATTAAAAATTTTAAGGCAAAAATC |
| 7693 cmeRAIVS (Type 0) | TTAAGTAAATATAAAAATTATTAACCAAATTTGAGCTATAATATGTCTTAAAAATTGTGTAATAAATATTACAATATATTTAAATTAAAAATTTTAAGGCAAAAATC |
| 7696 cmeRAIVS (Type 0) | TTAAGTAAATATAAAAATTATTAACCAAATTTGAGCTATAATATGTCTTAAAAATTGTGTAATAAATATTACAATATATTTAAATTAAAAATTTTAAGGCAAAAATC |
| 7700 cmeRAIVS (Type 0) | TTAAGTAAATATAAAAATTATTAACCAAATTTGAGCTATAATATGTCTTAAAAATTGTGTAATAAATATTACAATATATTTAAATTAAAAATTTTAAGGCAAAAATC |
| 7702 cmeRAIVS (Type 0) | TTAAGTAAATATAAAAATTATTAACCAAATTTGAGCTATAATATGTCTTAAAAATTGTGTAATAAATATTACAATATATTTAAATTAAAAATTTTAAGGCAAAAATC |
| 7704 cmeRAIVS (Type 0) | TTAAGTAAATATAAAAATTATTAACCAAATTTGAGCTATAATATGTCTTAAAAATTGTGTAATAAATATTACAATATATTTAAATTAAAAATTTTAAGGCAAAAATC |
| 7711 cmeRAIVS (Type 0) | TTAAGTAAATATAAAAATTATTAACCAAATTTGAGCTATAATATGTCTTAAAAATTGTGTAATAAATATTACAATATATTTAAATTAAAAATTTTAAGGCAAAAATC |
